# A bioinformatic pipeline to analyze ChIP-exo datasets

**DOI:** 10.1093/biomethods/bpz011

**Published:** 2019-08-06

**Authors:** Christoph S Börlin, David Bergenholm, Petter Holland, Jens Nielsen

**Affiliations:** 1 Department of Biology and Biological Engineering, Chalmers University of Technology, Gothenburg, SE-41296, Sweden; 2 Novo Nordisk Foundation Center for Biosustainability, Chalmers University of Technology, Gothenburg, SE-41296, Sweden; 3 Novo Nordisk Foundation Center for Biosustainability, Technical University of Denmark, Kgs. Lyngby, DK-2800, Denmark; 4 BioInnovation Institute, Ole Maaløes Vej 3, DK2200, Copenhagen N, Denmark

**Keywords:** chromatin immunoprecipitation, analytical pipeline, ChIP-exo

## Abstract

The decrease of sequencing cost in the recent years has made genome-wide studies of transcription factor (TF) binding through chromatin immunoprecipitation methods like ChIP-seq and chromatin immunoprecipitation with lambda exonuclease (ChIP-exo) more accessible to a broader group of users. Especially with ChIP-exo, it is now possible to map TF binding sites in more detail and with less noise than previously possible. These improvements came at the cost of making the analysis of the data more challenging, which is further complicated by the fact that to this date no complete pipeline is publicly available. Here we present a workflow developed specifically for ChIP-exo data and demonstrate its capabilities for data analysis. The pipeline, which is completely publicly available on GitHub, includes all necessary analytical steps to obtain a high confidence list of TF targets starting from raw sequencing reads. During the pipeline development, we emphasized the inclusion of different quality control measurements and we show how to use these so users can have confidence in their obtained results.

## Introduction

Transcription factors (TFs) are one of the main determinants of transcriptional regulation and therefore there has been much interest in precisely mapping their binding sites to identify their regulatory targets. To this date, several methods have been developed to map TF binding sites in the genome. Most of these were based on chromatin immunoprecipitation (ChIP), a method in which antibodies are used to selectively enrich for a certain TF or chromatin feature. The first usage of ChIP for genome-wide mapping was ChIP-chip, using DNA microarrays to quantify the bound sequences [1]. Shortly afterwards the method was upgraded to utilize the available sequencing technology to increase the quality of obtained data and to be independent on predefined sequences on the DNA microarrays (ChIP-seq) [2]. In 2011, this method was further refined by Rhee et al. using an additional exonuclease treatment following the immunoprecipitation (chromatin immunoprecipitation with lambda exonuclease; ChIP-exo), to improve the resolution of the mapped TF peaks to the single nucleotide level and to increase the signal-to-noise ratio (SNR) of the method [3]. An updated protocol simplifying the method was published in 2018 (ChIP-exo 5.0) [4].

Even though the original ChIP-exo method is now available for nearly 7 years, so far, no complete bioinformatics pipeline for analyzing the data is publicly available. Here we demonstrate such a pipeline, starting from the raw sequencing files, to identified peaks and gene targets. This pipeline stitches together heavily used software tools from other parts of the genomic research areas, like Bowtie2 for mapping the sequencing reads [5], and a peak finding tool developed for ChIP-seq and ChIP-exo called Genome wide Event finding and Motif discovery (GEM) [6]. The outputs of the software tools are analyzed using custom designed scripts written in Python to ensure a wide applicability of our pipeline. This pipeline is only for ChIP-exo data and not applicable for ChIP-seq data as there are fundamental differences about how ChIP-seq and ChIP-exo data should be handled. The pipeline also includes analytical steps leveraging the high resolution of ChIP-exo which would not be possible with ChIP-seq data.

## Materials and methods

### Mapping of sequencing reads

The obtained paired-end sequencing reads where mapped to the previously published CEN.PK113-7D genome [7] using bowtie2 [5]. The settings –no-mixed and –no-discordant were used to ensure that only read pairs where both reads map together where kept.

### Filtering of PCR duplicates

During library preparation of the ChIP-exo samples, PCR amplification is used which can result in PCR duplicates. PCR duplicates are defined as a set of read pairs, that all have an identical read_1 and also an identical read_2 (see also Rossi et al.[4]). The thought behind this definition is that only the position of read_1 is clearly defined by the exonuclease treatment, while the position of read_2 is caused by the random fragmentation of the DNA through sonication. Therefore, there are many read_1’s that have identical positions, but the chance of obtaining the same read_2 position is so small that one can assume that any duplication observed was caused by the PCR amplification. To find and exclude these PCR duplicates, samtools was used [8] using the following steps: filter out low-quality (<20) reads, sort reads by name (sort -n), fix read pairs (fixmate), sort reads by chromosomal position (sort), mark duplicates (markdup), and finally extract only read_1 from each nonduplicated pair (view -f 0x40).

### Determining optimal trim length

The main difference of ChIP-exo to ChIP-seq is that the obtained sequence reads mark the boundaries of the TF binding site. To ensure that the reads from both sides will overlap and therefore mark the position of the TF binding site, one has to select an appropriate trim length. The optimal trim length for each TF will be different, because TFs have different sizes and consequently differently sized footprints on the DNA. Therefore, we developed a set of mathematical formulas to convert the sequence length of a TF into an approximate footprint size, as shown below:

$$T\mathrm{Fweight}=\mathrm{sequenceLength}\;\left[\mathrm{nuc}\right]\;\text{×}\;\frac{1\;\mathrm{AA}}{3\;\mathrm{nuc}}\;\text{×}\;110\frac{\mathrm{daltons}}{\mathrm{AA}}$$

$$\mathrm{TFradius}=0.066\;\frac{\mathrm{nm}}{\mathrm{daltons}}\;\text{×}\;\;{\mathrm{TFweight}\;\left[\mathrm{daltons}\right]}^{\frac{1}{3}}$$

$$\mathrm{TFfootprint}=3\;\text{×}\;\mathrm{TFradius}\;\text{×}\;3.03\frac{\mathrm{bp}}{\mathrm{nm}}$$

First, the weight of the TF is calculated based on an average amino acid weight of 110 daltons. Then the radius of the TF is estimated based on the assumption that the shape is spherical (see Erickson [9]). The majority of TFs bind as dimers (either homo or hetero) and we make the assumption that the dimers overlap for half their size. Therefore, we take 3 times the radius of the TF and convert that size into number of bps. After rounding, this results in the optimal trim length in bp.

### Creating read profile files

The reads determining the border positions of the TF have to be converted into the binding profiles. For this step, first the “genome coverage” function of BEDTools [10] is used to generate the read profiles for both strands separately using the determined optimal trim length. Subsequently, both strands are combined and only base position where there are reads on both strands are reported as the TF binding profile. After this step, the replicates are normalized based on their average background read count and then combined using their average read count per base position.

### Peak detection

To detect the TF peaks, the software GEM [6], version 3.4, was used on the trimmed bam files (trimming was performed using bamUtils [11]). The following parameters where set: *q*-value threshold of 0.01, length of kmer between 5 and 18, smoothing width of 3, minimum number of events of 5 and a maximum read count per base position of 50. For each detected peak, the measured IP strength was normalized to the expected strength at that position and peaks with a resulting SNR ≤2 were filtered out.

The resulting peaks where mapped to genes based on their distance to the transcription start site (TSS), which were obtained from Börlin et al. [12]. Peaks that have their center point <1000 bp away from a TSS annotation (independent of upstream of downstream) are assigned to the corresponding gene. If a peak is close enough to two genes, it will be assigned to both genes.

### Motif discovery

For motif discovery, the 60 bp sequence underneath the detected GEM peaks (centered on the GEM peak position) were extracted using the “getfasta” function from BEDTools and analyzed using MEME [13]. The following settings for MEME were used: classic objective function, size of motif between 5 and 20, search mode zoops, 0-order Markov model, identify up to three motifs, also search for reverse complementary motifs.

### Creating an artificial dataset with increasing levels of noise

For creating an artificial dataset with defined levels of added noise, the tool ArtificialFastqGenerator [14] was used to create 75 bp paired-end reads with an average (SD) insert length of 225 (75) (the values were chosen to match the Ino2 sequencing data). The reads were mapped to the genome using the same steps as for the Ino2 data to create a randomly distributed set of reads.

Both replicates of Ino2 in the glucose-limited condition were merged together using “samtools merge” to create a unified set of Ino2 reads.

Following this, five different mixed datasets with specified levels of noise (0, 25, 50, 75, and 100%) were created with 100.000 reads each by subsampling from the two datasets (Ino2 and the artificial data) using the “samtools view -s” command. The number of reads that were randomly selected for each noise level is shown in Table 1.

**Table 1:** Number of reads subsampled for creating the mixed datasets

Noise level (%)	Number of Ino2 reads sampled	Number of artificial reads sampled
0	100.000	0
25	75.000	25.000
50	50.000	50.000
75	25.000	75.000
100	0	100.000

For each noise level, two independent samples were created to mimic biological duplicates. For each call of the “samtools view -s” command, a different seed was used to ensure independent sampling.

## Results and discussion

**Figure 1:**
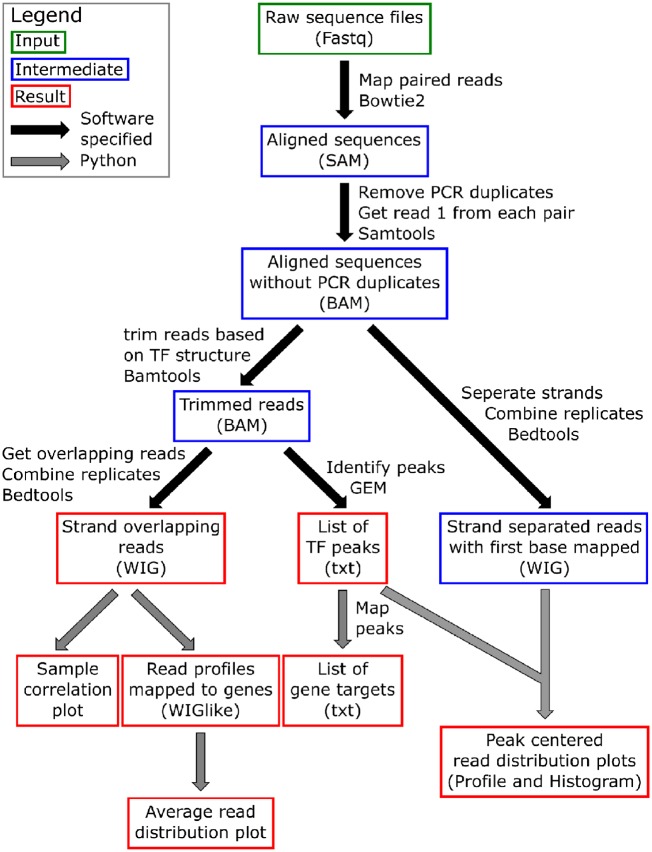
Overview of bioinformatics pipeline for ChIP-exo data. Here all steps are shown, with computational steps represented as arrows and boxes for files. The pipeline starts with a number of computational steps using existing software tools (marked with black arrows), producing intermediate results files (marked with blue boxes). The final analysis of the output is done using custom made python scripts (marked with gray arrows) producing the final output files (marked with red boxes).

Here we show the results from the analysis pipeline run on ChIP-exo sequencing data of Ino2 in two different conditions; respiratory glucose metabolism using glucose limitation (abbreviated as Glu) and gluconeogenic respiration using ethanol limitation (abbreviated as Eth). The sequencing data were previously published in Bergenholm et al. [15]. A detailed overview of each individual step in the pipeline, as well as which program or script was used is shown in Fig. 1.

In short, the pipeline starts with mapping the sequencing files to the genome and subsequently removes PCR duplicates and converts it into .bam files. Based on the predetermined optimal trim length, determined by the sequence length of the TF, the .bam files are trimmed and subsequently converted into wig files. In the conversion process to the wig files, read positions that do not overlap any read on the other strand are filtered out. The reasoning behind this is that ChIP-exo defines both borders of the TF, one on the forward strand and one of the reverse strand, with the TF sitting in between. One can only be confident in the presence of a TF if both borders are mapped with sequencing reads and overlap in the middle, which is then the actual location of the TF.

These wig files are the base for most of the subsequent analysis we show here, they also have the added benefit that they are easily visualized in a genome browser like the Integrated Genomics Browser [16].

To make it easier for the reader to follow, the section about the output of the pipeline will be split into two parts: (i) the text file outputs and (ii) the graphical outputs.

### Text file outputs

The pipeline has two major text file outputs, the gene target list and the GEM analysis file. The gene target list is a csv file that shows which genes are targeted by the selected TF in which condition. This is done by displaying the number of peaks on each gene in each condition, a gene is considered a gene target if it has at least one TF binding peak. With this file, one can easily run a gene set enrichment analysis to get further insight into which genes are targeted and how that changes between the different conditions. Therefore, this is the most important output of the pipeline and provides the basis for most subsequent analysis the user can perform on their own.

The other text file, the GEM analysis file, gives more detailed about each individual detected peak that could be assigned to a gene (that means the peak center was <1000 bp away from the gene’s TSS). Here each peak is listed with its strength and position, as well to which gene it was assigned. With this, one can compare more in detail how different genes are targeted by the selected TFs and check if there are correlations between TF binding strength and the response in expression levels.

### Graphical output

The pipeline produces four different graphical outputs, which will be described in detail together with instructions on how to use and analyze them.

**Figure 2:**
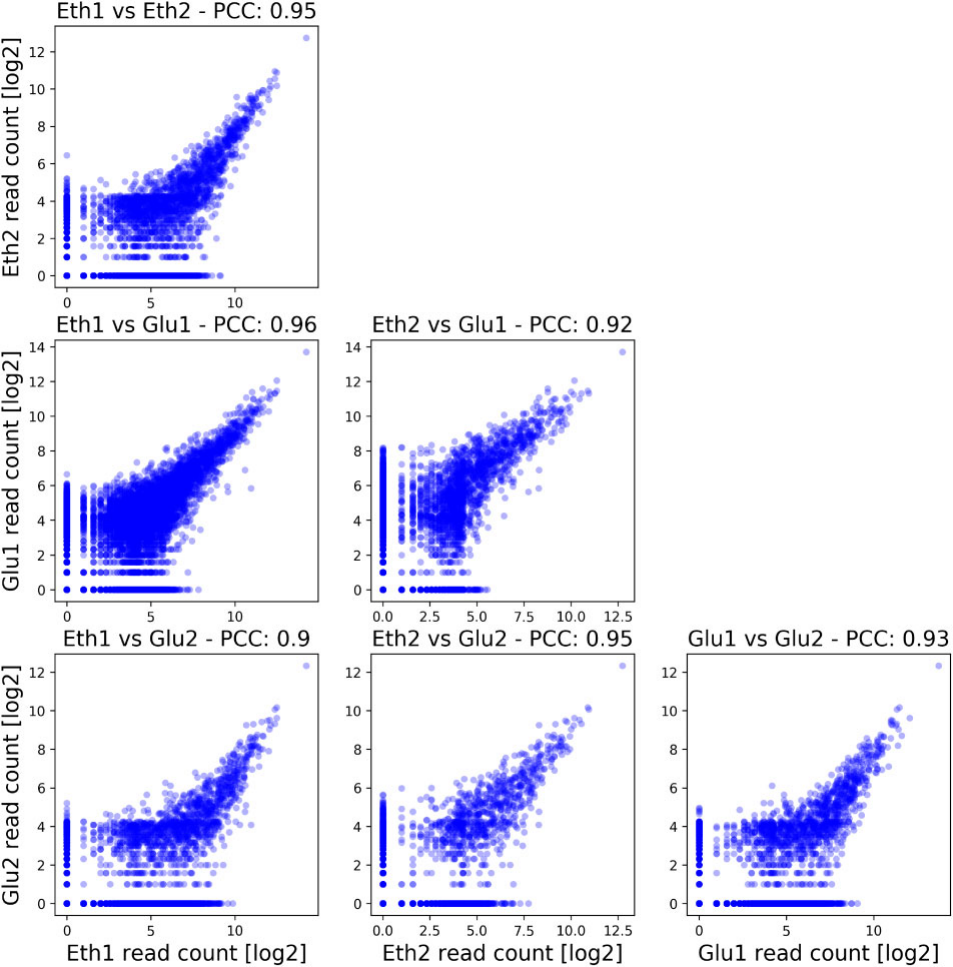
Pairwise comparison of all four samples. This automatically generated output figure shows the correlation between each individual sample using genomic bins of 1 kb size. The Pearson correlation coefficient is also shown in the title of each plot. This result shows a very high correlation between the four samples in the study, highlighting the quality and low level of noise of the ChIP-exo method.

The first plot produced is the sample correlation plot, which main function is to serve as quality control, as one can easily check if the replicates show a high correlation to each other. It is produced by binning all the reads over the whole genome into bins of 1 kb size. This binning strategy greatly reduces the number of variables to a manageable number and smoothens out noise in individual locations. Bins that had zero reads in all samples were filtered out and the remaining binned value for each replicate where log_2_ transformed and plotted against each other. In addition, the Pearson correlation coefficient was calculated and shown in the graphical output (Fig. 2).

In the example dataset for Ino2, one can observe that the replicates for both conditions show a high correlation to each other, highlighting the high quality of the sequencing data. Due to low noise sensitivity of the ChIP-exo method, replicates should show a Pearson correlation coefficient of at least 0.85 to each other, good replicates have ≥0.9. Both conditions also show a strong correlation to each other, hinting that the underlying regulatory program and therefore the targets of Ino2 are very similar in both these respiratory conditions.

**Figure 3:**
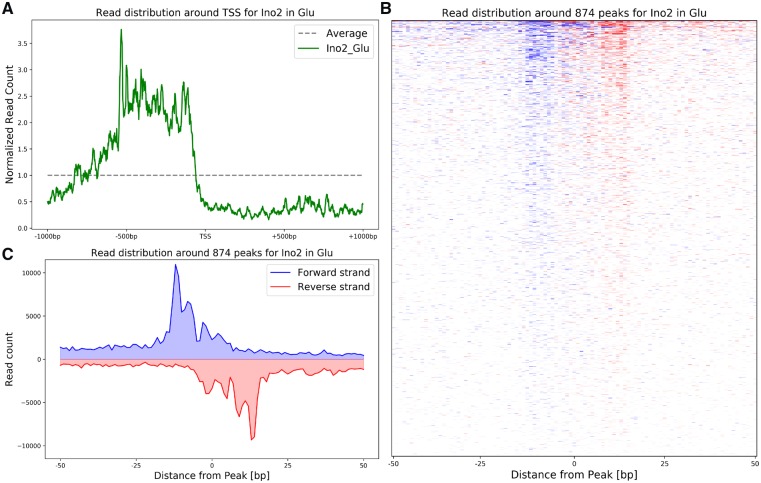
Overview of the pipeline output figures. (**A**) In this plot, the overall read count distribution (coming from overlapping reads on both strands) is shown across the promoter region. One can observe an enrichment of reads upstream of the TSS. (**B**) This histogram shows the read distribution (with only the first base of each read mapped to the genome) around all 874 peaks for Ino2 in glucose-limited conditions on an individual peak level, where every line corresponds to a single peak. (**C**) This plot shows the average read distribution from all the Peaks shown individually in B.

The second plot produced is showing the overall distribution of reads around the TSS using the previously created read profiles. For this, all positions ±1000 bp of each TSS where averaged, normalized to the average background read count across the genome and then plotted (Fig. 3A). This plot also serves as a quality control, as it is known that most TFs bind in the region upstream of the TSS up to 750–1000 bp away in yeast. Therefore, one would expect an enrichment of reads in this region in comparison to the average, which is exactly what one can observe in the example data (Fig. 3A).

The last two plots enable analysis of the peak shape of the mapped TF. For this, the single nucleotide read profile on both strands around each TF peak (as identified by GEM) was plotted as a line in the peak profile histogram (Fig. 3B). All individual peak profiles were also averaged and plotted together (Fig. 3C). These two plots show the identified borders of the TF and can therefore also serve as a quality control. Ideally, one would like to obtain a histogram with exactly one fine blue line and one fine red line, showing that the distance from the peak center to the borders is identical for each individual peak. In reality, this perfect border will be very difficult to obtain, and the norm would be to have a clearly defined peak on both strands with some smear to the sides. The results in Fig. 3B and C show this smeared behavior and we would still classify this as a very clear result. If the peak detection would have failed or the peaks being of very different size and shape, one would expect a much wider smear, completely obscuring the single peak. In the example data, one can see that the highest peak is at 11–13 bp away from the center point for both the plus strand (shown in blue) and the minus strand (shown in red). This resulting 22–24 bp window between the two peaks matches very well to the calculated footprint of 19 bp using the above described formula, demonstrating that this approximation is sound. The footprint calculation is influenced by the shape and therefore also by the class of the TF in question. In Supplementary Fig. S1, we show the results for two other classes of TFs, the zinc clusters and the basic leucine zippers, with high-quality results. In addition, we also show that the method does not only work on Ino2 as the example for a basic helix–loop–helix TF but show one more TF of this class. Taken together, we are confident that our footprint calculation method is valid for all classes of TFs in yeast.

All in all, the main purpose of the different graphical outputs is more quality control than generating actual biological insights. However, with ensuring high quality through the graphical outputs, one can place more trust in the raw data and the obtained target lists.

### Motif discovery

**Figure 4:**
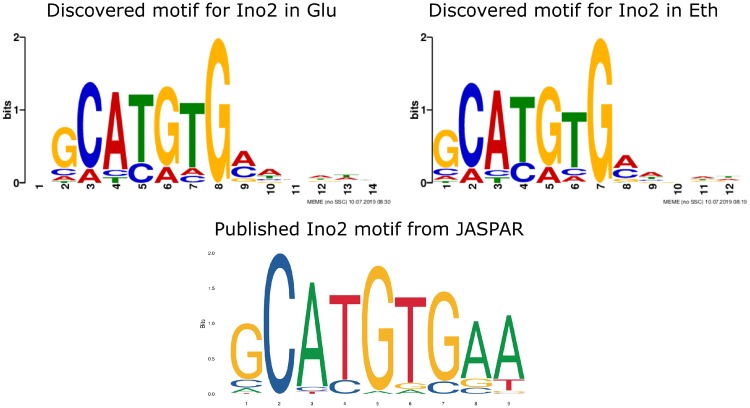
Motif comparison for Ino2. The upper row shows the discovered motifs by MEME, on the left in the glucose-limited condition, on the right in the ethanol-limited condition. For comparison, the published motif obtained from the JASPAR database is shown in the bottom row.

As part of the pipeline, enriched motifs in the peaks are detected using MEME. Running this part of the pipeline will create an output folder specific for MEME including the html report. In this report, one can see which motifs are detected in how many of the sequences. In addition, one can use the detailed text output files to find which sequences exactly are bound. The most enriched detected motif for Ino2 in both conditions is shown in Fig. 4. The published Ino2 motif obtained from the JASPAR database [17] is also displayed in Fig. 4, showing a high degree of similarity, validating the pipeline. The same approach was used for the three other TFs used in this study, Stb5, Gcn4, and Cbf1, and their discovered motifs in comparison to the published motifs are shown in Supplementary Fig. S2.

### Validating the noise robustness of the pipeline on artificial mixed data

To assess the robustness of the pipeline to noisy datasets and to further demonstrate how the graphical outputs can be used for quality control, we created five artificial datasets, mixing the real Ino2 data with artificial created sequencing reads (corresponding to noise) with 0, 25, 50, 75, and 100% noise as described in the materials and methods section.

**Figure 5:**
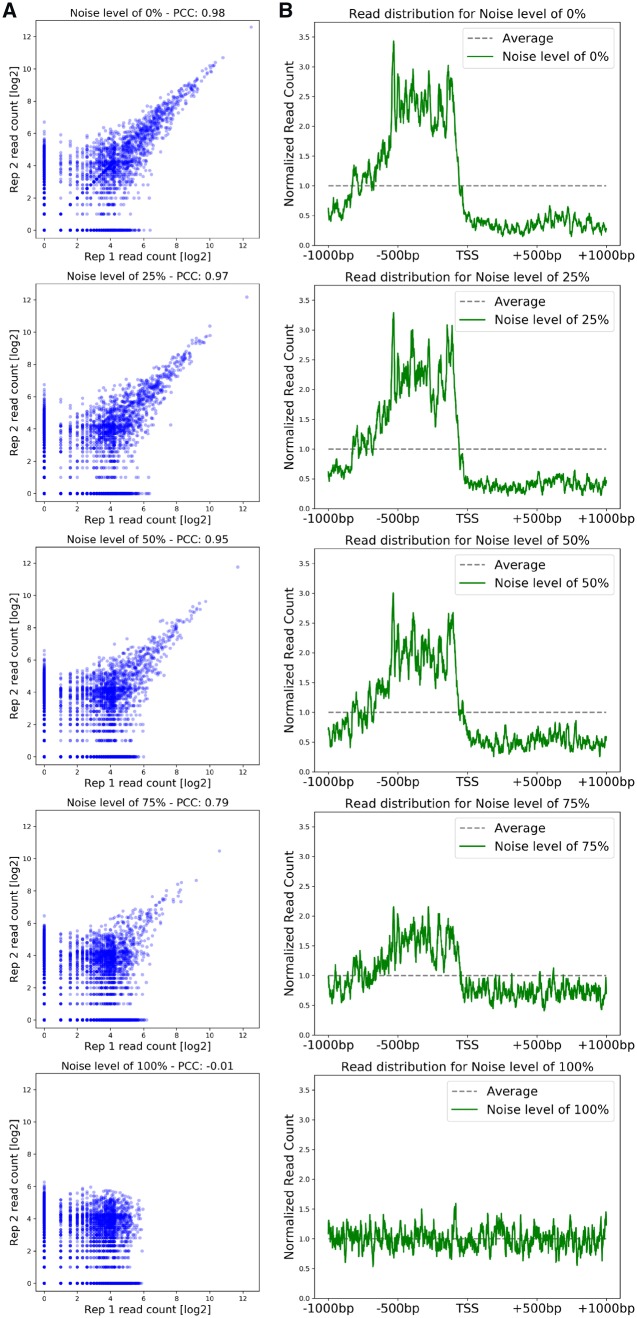
Comparison of artificial noisy data for robustness. (**A**) In this panel, the duplicates for each different noise level are compared and the Pearson correlation coefficient is shown. (**B**) In this panel, the read distribution around the TSS is shown for each of the five different noise levels.

The pipeline was run on these 10 artificial samples (two for each noise level) and the correlation between the duplicates of each noise level is shown in Fig. 5A. The full pairwise correlation plot can be found in the Supplementary Fig. S3. The Pearson correlation coefficient between the samples decreases with increasing level of added noise as expected and shows a strong drop in the samples with 75% noise. The pearson correlation coefficient (PCC) of 0.79 is below the threshold of 0.85, the limit of what we would consider acceptable. The 100% noise sample shows no correlation as one would expect in two completely randomized “replicates.” In Fig. 5B, the read profile for each noise level is shown and here one can also observe that the enrichment in the promoter region decreases with increasing level of noise. The samples with 0, 25, and 50% of noise still show a strong enrichment while the 75% noise sample only shows a very weak enrichment and the 100% noise sample shows no enrichment.

Based on the low PCC between the replicates in the 75% noise sample as well as the weak enrichment of the reads in the promoter region, we would consider this a bad dataset which is too noisy for further analysis, while the data with 0, 25, and 50% noise would pass our quality control steps.

## Conclusion

In this protocol, we comprehensively show how to analyze ChIP-exo data starting from raw sequencing results. The pipeline, which is freely available at GitHub, produces a number of graphical output files for quality control, as well as an easy to use gene target list. Therefore, the pipeline provides the user with a strong basis for further analysis of the TF binding behavior.

### Data and code accessibility

The complete pipeline is hosted at Github: https://github.com/SysBioChalmers/ChIPexo_Pipeline

The ChIP-exo data for Ino2 in the two conditions shown here is directly accessible through Zenodo: https://doi.org/10.5281/zenodo.3242510

The full ChIP-exo dataset (including two more conditions) is accessible at the Gene Expression Omnibus (GEO) under the accession number GSE88941 (https://www.ncbi.nlm.nih.gov/geo/query/acc.cgi? acc=GSE88941).

## Supplementary data

Supplementary data is available at *Biology Methods and Protocols* online.

## Funding

This work was supported by the European Union’s Horizon 2020 research and innovation program [Marie Skłodowska-Curie grant agreement No 722287], the Knut and Alice Wallenberg Foundation and the Novo Nordisk Foundation [grant number NNF10CC1016517].

## Supplementary Material

bpz011_Supplementary_Data

## Appendix

How to setup the pipeline to run it on the provided example data:

1. Clone/download the code from Github (https://github.com/SysBioChalmers/ChIPexo_Pipeline)
2. Download the Ino2 data from Zenodo (https://doi.org/10.5281/zenodo.3242510) in to the Data folder, filename should be Ino2_rawdata.tar.gz.
3. In the script update the paths to Bowtie2, samtools, BEDTools and bamUtils so that they can be added to the $PATH Linux variable, if they are not already added and callable (Lines 26–30).
4. Set the paths for the output and data files if needed (Lines 13–23). The basic setup is using relative path that does not need to be changed.
5. Select which parts of the pipeline should be run (Lines 40–48) and how many cores are available for computation (Line 50).
6. To run it, use a Linux terminal, navigate to the main folder where the ChIPexo_Pipeline_MAIN.bash file is located and execute it using “bash ChIPexo_Pipeline_MAIN.bash.”

How to run it for a different TF or condition:

1. Change the variable TF in Line 33 and/or variable condList in Line 37 of the ChipExo_Pipeline_MAIN.bash file
2. Create a file called TFName_sequenceLength.txt to replace Ino2_sequenceLength.txt in the Data folder specifying the sequence length of the chosen TF. The file should contain one line “seqLength=X” where X is the actual sequence length in bp of the chosen TF.
3. Create a file called TFName_seqFiles.txt to replace Ino2_seqFiles.txt in the Data folder. In this file, the names of the sequencing files for each sample should be specified for Read 1 and Read 2. If multiple files should be used one can separate the files using a colon [e.g. seqFiles(“NewTF_NewCondition_R1”)=“File1_R1.fastq.gz, File2_R1.fastq.gz”].

How to change to another organism/genome version:

To run the pipeline for another organism or genome version, the following files are needed to replace the CENPK113-7D files distributed with the pipeline:

1. Update the variable refGenomeName in Line 22 of the ChipExo_Pipeline_MAIN.bash file to the name of the new reference genome
2. Genome sequence as a fasta file to replace file CENPK113-7D.fasta in Data/RefGenomeBowtie
3. Bowtie2 index of the genome to replace CENPK113-7D.XXX files in Data/RefGenomeBowtie
4. Sequence of each individual chromosome as a fasta file starting with chr for GEM, replacing the files in Data/RefGenome
5. Text file with the length of each chromosome, replacing CENPK113-7D_chromSizes in Data/RefGenome
6. If GEM should filter out a certain region of the genome, this needs to be specified in the file GEMexclude.txt in Data/RefGenome
7. If the pipeline should filter out a certain region of the genome or whole chromosomes (e.g. the mitochondria) this needs to be specified in the files Filterlist_regions.txt and Filterlist_chromosomes.txt in the Data folder.
8. TSS annotations to replace TSSData.tsv in the Data folder. This file should be tab separated and list a TSS including the strand for each gene. It has to include these four columns in this order: name of the gene, chromosome, TSS position and strand.
