# Supplementary material for: A bioinformatic pipeline to analyze ChIP-exo datasets
Source: Biol Methods Protoc. 2019 Aug 6;4(1):bpz011. doi: 10.1093/biomethods/bpz011 (PMC7200897; doi:10.1093/biomethods/bpz011)
Supplement: bpz011_Supplementary_Data [file bpz011_supplementary_data.docx]

A bioinformatic pipeline to analyze ChIP-exo datasets

Authors:

Christoph S. Börlin^1^, David Bergenholm^1^, Petter Holland^1^, Jens Nielsen^1,2,3,4*^

Affiliations:

1: Department of Biology and Biological Engineering, Chalmers University of Technology, Gothenburg, SE-41296, Sweden

2: Novo Nordisk Foundation Center for Biosustainability, Chalmers University of Technology, Gothenburg, SE-41296, Sweden

3: Novo Nordisk Foundation Center for Biosustainability, Technical University of Denmark, Kgs. Lyngby, DK-2800, Denmark

4: BioInnovation Institute, Ole Maaløes Vej 3, DK2200 Copenhagen N, Denmark

To whom correspondence should be addressed. Tel: +46 031 772 3804; Fax: +46 031 772 3801; Email: nielsenj@chalmers.se

**SUPPLEMENTARY INFORMATION**


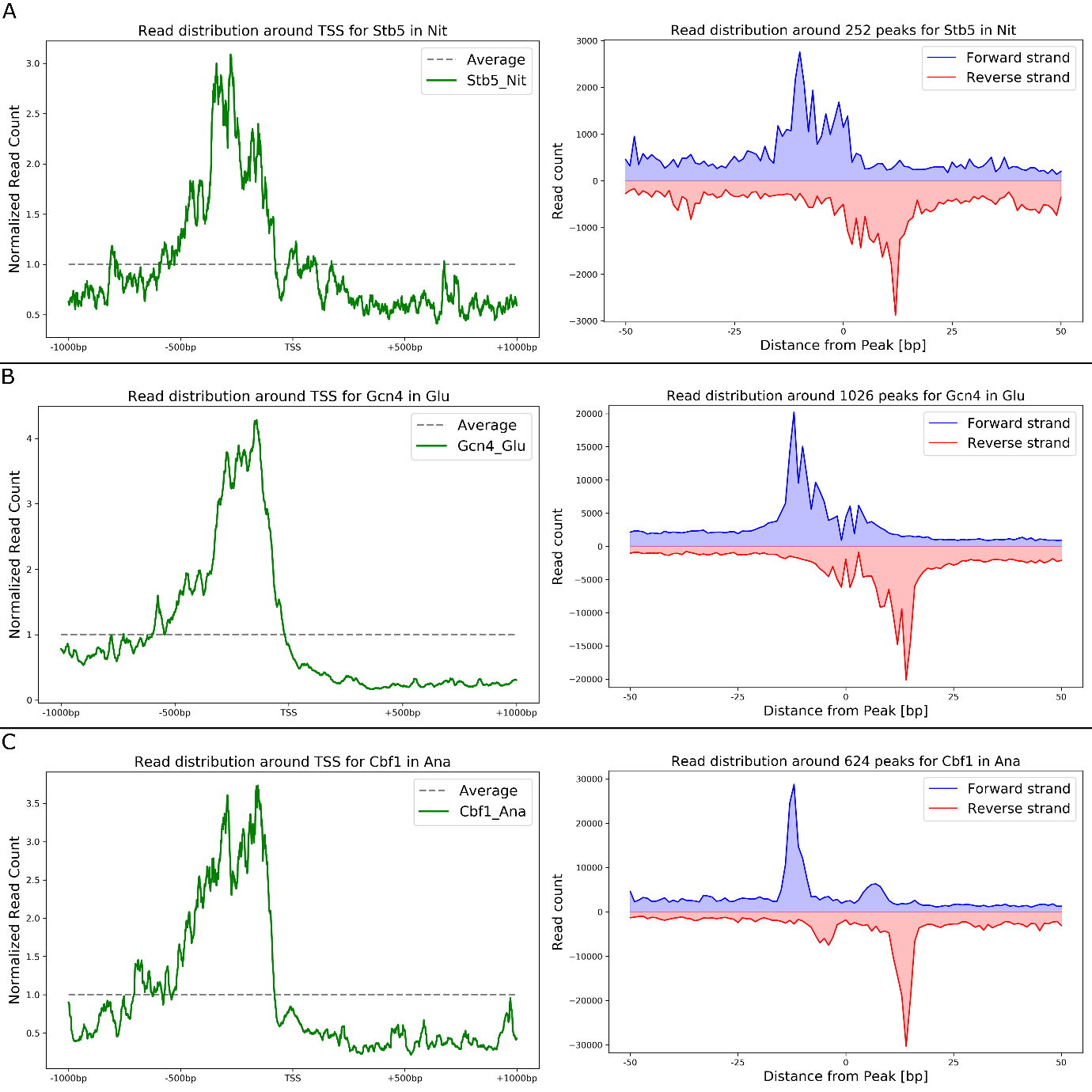


**Figure S1 Read distributions around TSS and detected Peaks for different classes of TFs.** **A** Showing the results for Stb5, a member of the zinc cluster TF family. **B** Showing the results for Gcn4, a member of the basic leucine zipper TF family. **C** Showing the results for Cbf1, a member of the basic helix-loop-helix TF family.

**
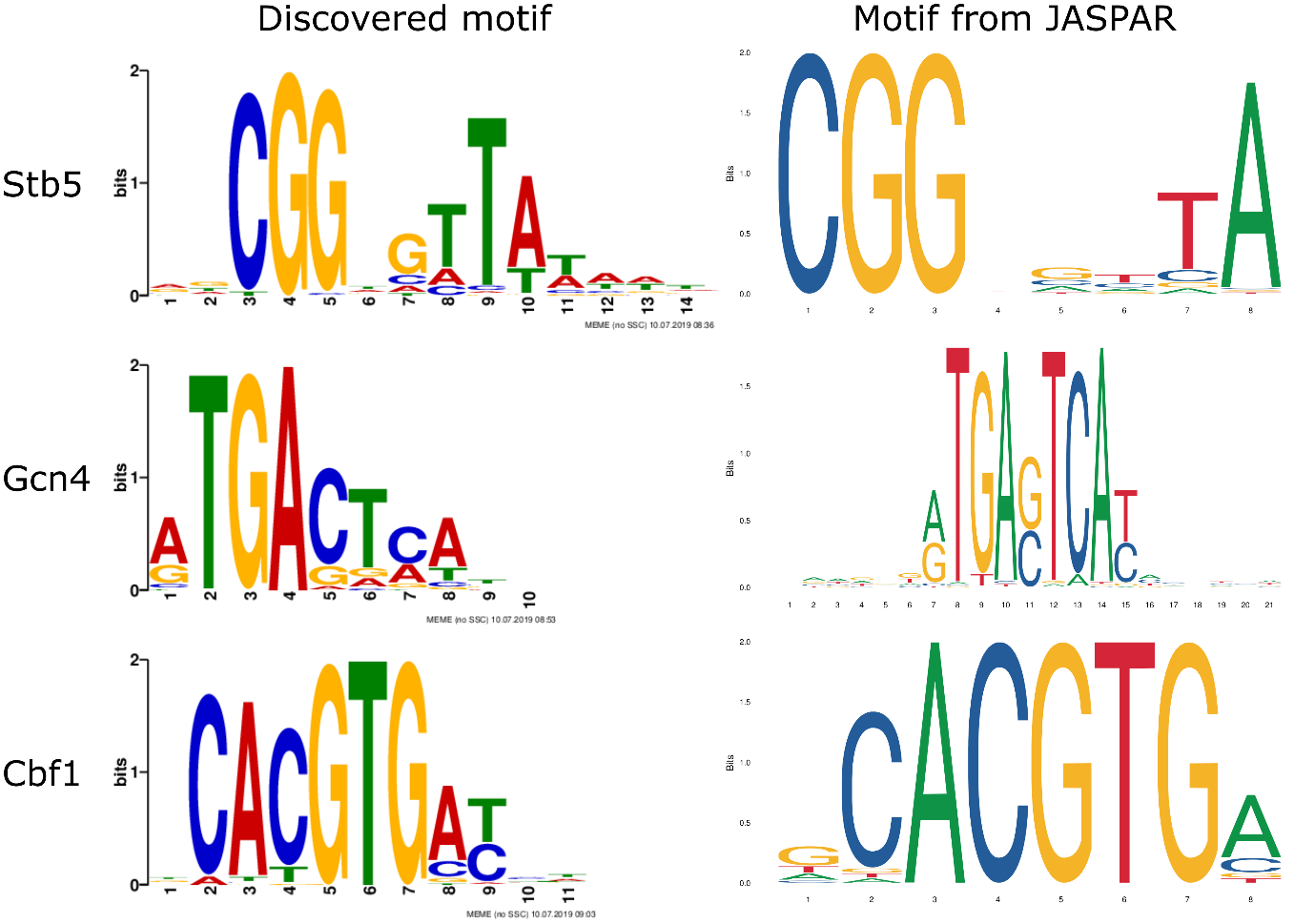
**

**Figure S2 Motif comparison for each additional TF shown.** On the left he discovered motif using MEME is shown for Stb5, Gcn4 and Cbf1. For comparison purposes on the right the motif for the same TF obtained from the JASPAR database is shown.

**
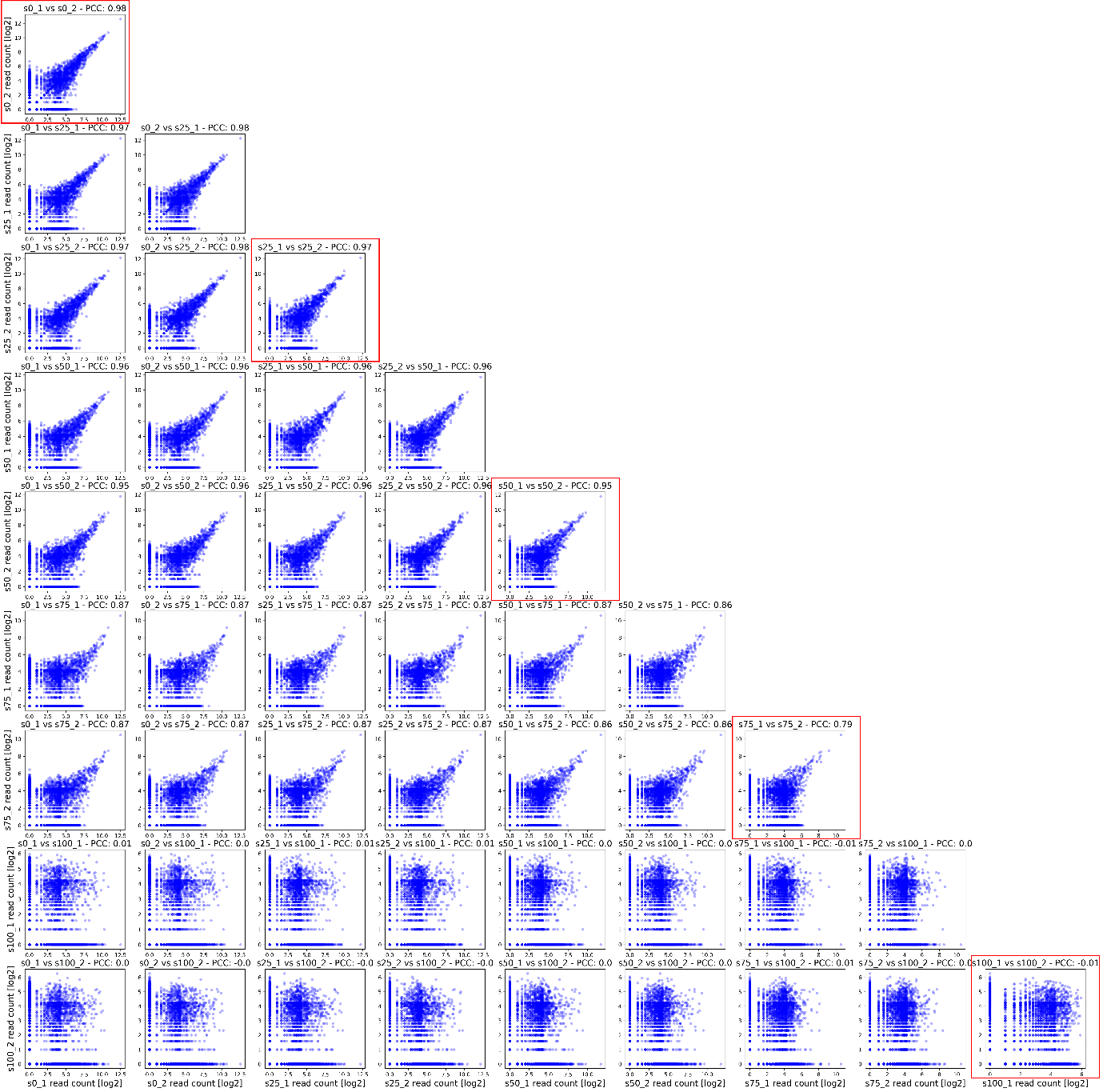
**

**Figure S3 Pairwise comparison of all 10 samples of the artificial dataset with increasing level of noise.** The Pearson Correlation Coefficient for each comparison is shown above each graph and the comparisons between the replicates are marked in red. The number after the s for each sample gives the amount of noise (from 0% to 100%) and the _1 and _2 in the end are to mark the two replicates.
